# Calcium-responsive transactivator (CREST) protein shares a set of structural and functional traits with other proteins associated with amyotrophic lateral sclerosis

**DOI:** 10.1186/s13024-015-0014-y

**Published:** 2015-04-10

**Authors:** Michail S Kukharsky, Annamaria Quintiero, Taisei Matsumoto, Koji Matsukawa, Haiyan An, Tadafumi Hashimoto, Takeshi Iwatsubo, Vladimir L Buchman, Tatyana A Shelkovnikova

**Affiliations:** School of Biosciences, Cardiff University, Museum Avenue, CF10 3AX Cardiff, UK; Institute of Physiologically Active Compounds Russian Academy of Sciences, 1 Severniy proezd, Chernogolovka, 142432 Moscow Region, Russian Federation; Department of Neuropathology, The University of Tokyo, Tokyo, Japan

**Keywords:** Amyotrophic lateral sclerosis (ALS), Calcium-responsive transactivator (CREST), SS18L1, Fused in sarcoma (FUS), TAR DNA‐binding protein 43 (TDP‐43), Protein aggregation, Stress granule, Neurodegeneration, Paraspeckle, Nuclear enriched abundant transcript 1 (NEAT1), Transgenic fly

## Abstract

**Background:**

Mutations in calcium-responsive transactivator (CREST) encoding gene have been recently linked to ALS. Similar to several proteins implicated in ALS, CREST contains a prion-like domain and was reported to be a component of paraspeckles.

**Results:**

We demonstrate that CREST is prone to aggregation and co-aggregates with FUS but not with other two ALS-linked proteins, TDP-43 and TAF15, in cultured cells. Aggregation of CREST affects paraspeckle integrity, probably by trapping other paraspeckle proteins within aggregates. Like several other ALS-associated proteins, CREST is recruited to induced stress granules. Neither of the CREST mutations described in ALS alters its subcellular localization, stress granule recruitment or detergent solubility; however Q388stop mutation results in elevated steady-state levels and more frequent nuclear aggregation of the protein. Both wild-type protein and its mutants negatively affect neurite network complexity of unstimulated cultured neurons when overexpressed, with Q388stop mutation being the most deleterious. When overexpressed in the fly eye, wild-type CREST or its mutants lead to severe retinal degeneration without obvious differences between the variants.

**Conclusions:**

Our data indicate that CREST and certain other ALS-linked proteins share several features implicated in ALS pathogenesis, namely the ability to aggregate, be recruited to stress granules and alter paraspeckle integrity. A change in CREST levels in neurons which might occur under pathological conditions would have a profound negative effect on neuronal homeostasis.

**Electronic supplementary material:**

The online version of this article (doi:10.1186/s13024-015-0014-y) contains supplementary material, which is available to authorized users.

## Background

Amyotrophic lateral sclerosis (ALS) is a fatal adult-onset neurodegenerative condition characterized by aetiologically diverse pathomechanisms, which ultimately results in loss of upper and lower motor neurons, paralysis and death. In a rapidly growing group of genes mutated in ALS the most represented are the genes encoding proteins directly or indirectly involved in RNA metabolism [1-3]. Structural and functional studies of ALS-associated proteins and their disease-linked variants have significantly contributed to our current understanding of the mechanisms of disease development and progression. Characterization of pathological signatures for each ALS-associated protein is crucial in delineating common pathways in the disease pathogenesis and eventually understanding how altered metabolism of proteins with diverse functions results in the same clinical phenotype.

Recently, using exome sequencing in sporadic ALS trios Chesi and co-workers [4] have identified two mutations in the SS18L1 gene which encodes calcium-responsive transactivator (CREST) protein. Subsequently two additional mutations in CREST, this time in patients with familial form of ALS, were reported [5]. CREST is a nuclear protein discovered in 2003 in a screen for calcium-responsive genes involved in transcriptional activation [6]. The same year the gene encoding CREST was independently described as translocated in some cases of synovial sarcoma [7]. CREST is important for normal development of the nervous system, and CREST-deficient mice display defective dendritic branching, motor disturbances and early lethality [6]. Importantly CREST interacts with CREB-binding protein (CBP), a histone acetylase known for its neuroprotective properties [8]. In the original study CREST was also shown to bind chromatin remodeling proteins BAF250 and BRG-1 [6]. Subsequently it was demonstrated that CREST and a highly homologous protein, SS18, are dedicated subunits of chromatin remodeling complex Brg/Brm-associated factor (BAF), which is an important modulator of transcription of specific gene sets at various stages of neural development [9]. Mutations in components of the BAF complex have been identified in autism, schizophrenia and other neurodevelopmental disorders, arguing that its function is crucial for normal development of the nervous system [10,11].

Structurally, CREST consists of a C-terminal transactivation domain, which is characterized by low sequence complexity and satisfies the criteria for a prion-like domain [4,6]; an N-terminal autoregulatory domain, which suppresses transactivation at basal state [6]; a central methionine-rich domain, and so called multifunctional domain (MFD) implicated in the protein dimerisation, regulation of transactivation and subcellular localization of the protein [12]. In the nucleus, CREST was shown to be recruited to nuclear bodies of unknown origin [13]. More recently, CREST was identified in a screen for paraspeckle proteins [14].

The majority of ALS-associated proteins are characterized by high aggregation propensity, which is attributable to the presence of a prion-like domain in their structure [15]. The ability to aggregate reversibly is indispensable for their normal function in RNA-protein macromolecular complexes, such as RNA transport granules, stress granules, paraspeckles and Gems; at the same time, pathological aggregation of these proteins is also governed by prion-like domains [16,17]. Despite the confirmed presence of a prion-like domain in CREST structure, the aggregation propensity of wild type CREST and its ALS-associated variants has not been addressed. Furthermore, dysfunction of paraspeckles/paraspeckle proteins has recently emerged as possible pathogenic factor in ALS [18,19]. The role of CREST in the paraspeckle is not clear, nor is it known if the protein can be recruited to other RNP complexes. Thus far, it has been shown that an ALS-associated CREST mutation leading to the deletion of the 9 C-terminal amino acids, Q388stop [4], abolishes its binding to CBP, suggesting that the missing amino acids act as an interface for interaction between the two proteins [6]. Both Q388stop and a mutation in autoregulatory domain, I123M, reduce depolarization-induced branching in cultured neurons [4]. In vivo effects of the other two mutations involving MFD and methionine-rich domain [5] have not been examined.

Therefore, in current study we aimed to characterize the aspects of CREST structure and interactions relevant to ALS pathogenesis in vitro and in vivo, primarily its aggregation propensity and possible involvement in the formation of nuclear and cytoplasmic RNA granules.

## Results

### CREST protein is prone to form aggregates in the cell nucleus

**Figure 1:**
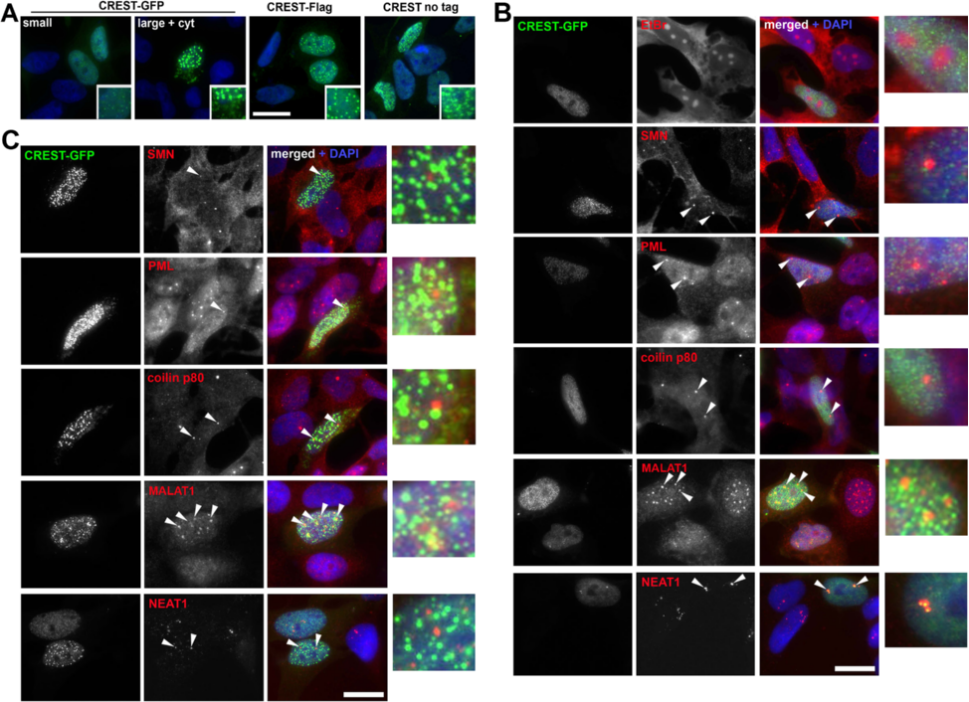
**CREST protein is aggregation-prone** ***in vivo*** **. (A)** CREST aggregates upon its accumulation in the nucleus. GFP-tagged, Flag-tagged or untagged CREST are largely confined to the nucleus where they are diffusely distributed or can form dot-like aggregates. In high-expressing cells CREST aggregates are visibly larger and the protein undergoes a shift to the cytoplasm where it also aggregates (‘large + cyt’ panel). SH-SY5Y cells expressing tagged or untagged CREST were analysed 24 hours post-transfection. **(B)** CREST-GFP is almost entirely confined to the nucleus where it is excluded from the nucleolus visualised by ethidium bromide (EtBr) staining. It is not enriched in PML bodies (anti-PML staining), Gems (anti-SMN staining) or Cajal bodies (anti-coilin p80 staining), but is concentrated around speckles (FISH with MALAT1 probe) and highly enriched in paraspeckles (FISH with NEAT1 probe) in low-expressing cells. **(C)** Nuclear aggregates formed by CREST-GFP overexpressed in SH-SY5Y cells do not overlap with PML bodies, Gems, Cajal bodies or paraspeckles but surround speckles. Arrowheads denote nuclear bodies in CREST-expressing cells. Scale bars, 10 μm.

Previous studies have demonstrated that in transfected cells exogenous CREST localizes to nuclear dot-like structures designated as nuclear bodies [12]; however their identity has not been determined. We generated constructs to express either untagged CREST or CREST tagged with GFP or Flag peptide. All three proteins displayed predominantly nuclear distribution in neuroblastoma SH-SY5Y and COS7 cells (Figure 1A, Additional file 1: Figure S1). In agreement with the results of the above study, we also observed formation of nuclear dot-like structures upon expression of tagged or untagged CREST (Figure 1A, Additional file 1: Figure S1). In cells with profound accumulation of CREST in the nucleus, the presence of the expressed protein in the cytoplasm and its cytoplasmic aggregation were also evident, particularly for CREST-GFP (Figure 1A, large + cyt panel, Additional file 1: Figure S1). To establish if nuclear dot-like structures formed by CREST were related to known nuclear bodies, transfected cells were co-stained for various nuclear body markers. In SH-SY5Y cells with a diffuse/fine-granular nucleoplasmic distribution of CREST-GFP the protein was excluded from nucleolar region identified by ethidium bromide staining, was not enriched in SMN-positive Gems, coilin p80-positive Cajal bodies or PML bodies but we detected its enrichment around MALAT1-positive nuclear speckles (Figure 1B). CREST was reported to be a paraspeckle component [14]; we also observed CREST-GFP enrichment in paraspeckles visualized by NEAT1 FISH, but only in cells with low levels and diffuse distribution of the protein (Figure 1B, bottom panel). Large dot-like nuclear structures formed by CREST did not overlap with any of the above nuclear bodies, including paraspeckles, though they often surrounded speckles (Figure 1C). Hereafter, these nuclear structures as well as cytoplasmic CREST accumulations of any appearance will be referred as “aggregates”.

**Figure 2:**
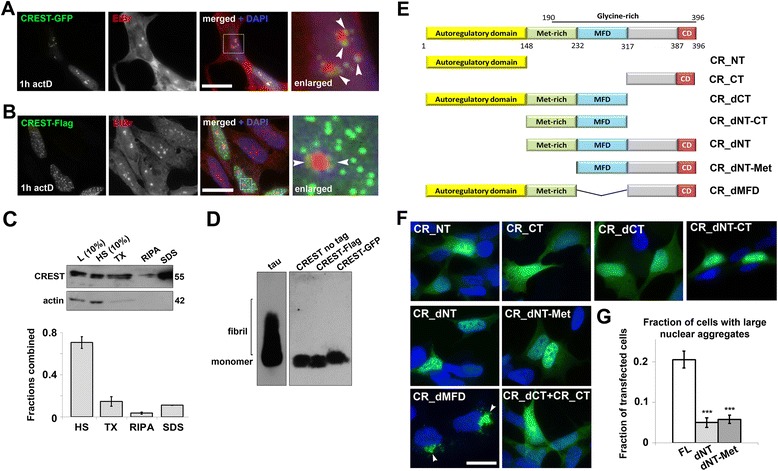
**Characterisation of the aggregation capacity of CREST. (A)** In cells with diffuse distribution of CREST, it re-localizes to nucleolar caps (arrowheads) in response to transcriptional inhibition. **(B)** The pool of CREST in nuclear dot-like aggregates fails to redistribute to nucleolar caps upon inhibition of transcription, and the aggregates persist under these conditions, although weakly CREST-positive nuclear caps can be observed (arrowheads). In A and B SH-SY5Y cells were exposed to actinomycin D for 1 hour. **(C)** CREST is recovered in detergent-insoluble fractions. HEK293 cells expressing untagged CREST were subjected to sequential protein extraction as described in Materials and methods. For total lysate (L) and high-salt (HS) fraction 10% of the amount relative to other fractions was loaded. Bar chart shows relative protein amounts (±s.e.m.) in each fraction quantified by densitometry. **(D)** Untagged CREST, Flag-CREST and GFP-CREST do not form SDS-resistant oligomeric forms. Cleared lysates of CREST-expressing SH-SY5Y cells were run in SDS-containing agarose gel; all variants were visualized using anti-CREST antibody. Mutant tau protein from spinal cord lysate of a transgenic P301S mouse (detected by phospho-tau-specific antibody) was used to demonstrate typical behavior of amyloid species in this assay. **(E)** Schematic representation of CREST deletion constructs used in the study. All variants were expressed as GFP-fusion proteins. **(F, G)** Distribution of CREST deletion mutants in SH-SY5Y cells. CR_dNT and CR_dNT-Met were shifted to the cytoplasm and formed nuclear dot-like aggregates less frequently than full-length protein (G). Bar chart in G shows the fraction of cells (mean ± s.e.m.) with nuclear aggregates for each variant (*** - p < 0.001; at least 150 cells counted per variant in each of the three independent experiments). CREST was expressed for 24 hours prior to actinomycin D exposure, cell lysis or fixation. Scale bars, 10 μm.

Consistent with the previous observation for Venus-tagged CREST [14], GFP-tagged, Flag-tagged and untagged CREST also redistributed to nucleolar caps in actinomycin D-treated cells expressing low levels of CREST (Figure 2A and data not shown). We argued that if dot-like CREST structures are stable, irreversibly aggregated entities they will not be affected by transcriptional arrest. Indeed, preformed nuclear aggregates of CREST remained intact following actinomycin D treatment (Figure 2B), although formation of weakly CREST-positive nuclear caps could be observed, probably due to protein redistribution from the pool of not yet aggregated protein (Figure 2B, arrowheads). Using live cell imaging, we also demonstrated that nuclear CREST aggregates are characterized by time- and concentration-dependent fusion and growth (Additional file 2: Video S1).

To explore CREST aggregation biochemically, we expressed untagged protein with subsequent sequential extraction in detergent-containing buffers (see Methods section for details). As shown in Figure 2C, approximately 10% of CREST remained in the TritonX-100/RIPA-insoluble fraction which could only be solubilised by boiling in the presence of 4% SDS. However, similar to some other ALS-associated proteins that form non-amyloid aggregates, neither tagged nor untagged CREST formed SDS-resistant amyloidogenic species, as was evident from detection of only monomeric forms of the protein in semidenaturating detergent agarose gel (SDD-AGE) assay (Figure 2D).

To assess the contribution of different CREST domains to its aggregation propensity, we produced a set of deletion constructs tagged with GFP at their N-terminus (Figure 2E) and expressed them in SH-SY5Y cells (Figure 2F). N-terminal autoregulatory domain (CR_NT) or C-terminal part of CREST (CR_CT) in isolation did not aggregate and were present both in the nucleus and in the cytoplasm. Deletion of C-terminal part completely abolished the protein’s ability to aggregate. In contrast, CREST lacking the autoregulatory domain only (CR_dNT) or in combination with Met-rich domain (CR_dNT-Met) was distributed and aggregated in a very similar manner to the full-length protein, i.e. these variants were detected mainly in the nucleus where they formed multiple puncta (Figure 2F). However, for these two variants, cytoplasmic delocalisation was more pronounced and aggregation capacity was diminished compared to full-length CREST (Figure 2F,G). The middle region of CREST comprising Met-rich and MFD domains (CR_dNT-CT) was efficiently targeted to the nucleus where it accumulated inside the nucleolus. Deletion of MFD domain rendered the protein highly aggregation-prone, and this variant was found almost exclusively in the form of aggresome-like cytoplasmic aggregates (Figure 2F). Co-expression of two complementary variants both displaying diffuse cellular distribution, CR_dCT and CR_CT, was not sufficient to trigger aggregation (Figure 2F), suggesting that this process requires the presence of the domains in cis. Therefore, both N-terminal and C-terminal parts of CREST are required for efficient aggregation, while central MFD might limit it.

### CREST is recruited to stress-induced stress granules

**Figure 3:**
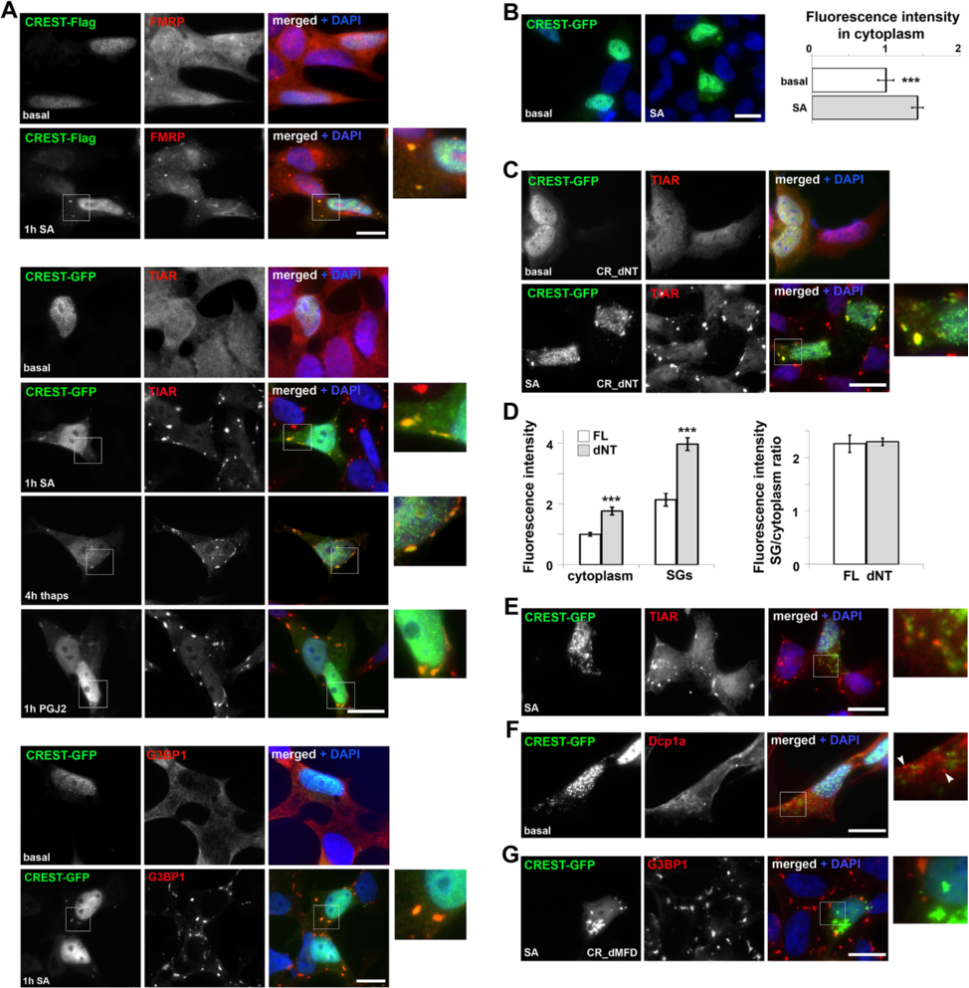
**CREST is targeted to stress granules by various stresses. (A)** CREST-Flag (top panel) and CREST-GFP (three bottom panels) are detected in stress granules induced by oxidative stress (sodium arsenite, SA), ER stress (thapsigargin, thaps) or inhibition of eIF4E (15d-PGJ2) and visualized with stress granule markers TIAR, FMRP and G3BP1. SA and 15d-PGJ2 were applied to SH-SY5Y cells for 1 hour and thapsigargin – for 4 hours. **(B)** In SH-SY5Y cells subjected to oxidative stress (SA for 1 hour) CREST-GFP undergoes significant shift to the cytoplasm. **(C, D)** CREST deletion mutant lacking autoregulatory domain (CR_dNT) is readily recruited to stress granules **(C)** and shows higher enrichment in these structures compared to full-length protein (**D**, left graph). This phenomenon is related to higher cytoplasmic levels of CR_dNT since the fluorescence intensity ratio stress granules/cytoplasm is similar for full-length and truncated protein **(D)**. **(E)** Cytoplasmic aggregates of CREST-GFP do not overlap with SA-induced stress granules but are found in their immediate vicinity. **(F)** Cytoplasmic aggregates of CREST-GFP do not overlap with P-bodies (visualized by anti-Dcp1a staining, arrowheads in the enlarged panel). **(G)** Aggresomes formed by GFP-tagged CREST lacking MFD domain are negative for a SG marker G3BP1. In **B** and **D**, fluorescence was measured in stress granules and/or cytoplasm of GFP-positive cells as described in Materials and methods, and cytoplasmic intensity for non-stressed cells **(B)** or full-length CREST-GFP **(D)** (mean ± s.e.m.) was taken as equal 1 (***p < 0.001). Scale bars, 10 μm.

A growing number of ALS-associated proteins have been shown to be the components of stress granules (SGs), cytoplasmic RNP foci assembled by cells in response to adverse conditions and facilitating translational shutdown under severe stress [20]. We examined CREST behaviour under various stresses and showed that CREST is also a resident of SGs. SH-SY5Y cells expressing full-length CREST-GFP or CREST-Flag were exposed to sodium arsenite, thapsigargin or a prostaglandin 15d-PGJ2 to trigger SG formation by inducing oxidative stress, ER stress or inhibiting translation elongation factor eIF4E [21], respectively. In cells with small aggregates or diffuse staining in the nucleus all three stimuli led to weak but reproducible CREST recruitment to SGs visualised with SG markers TIAR, G3BP1 or FMRP (Figure 3A), which was facilitated by the emergence of typically nuclear CREST in the cytoplasm of stressed cells (Figure 3B). Only two CREST deletion mutants – those lacking N-terminal domains, namely CR_dNT and CR_dNT-Met, – were recruited in SGs, moreover, they were detected at higher levels within SGs compared to the full-length protein (Figure 3C,D and data not shown). This was likely due to their cytoplasmic redistribution rather than enhanced propensity for SG recruitment since fluorescence intensity ratio SG/cytoplasm was similar for CR_dNT and full-length protein (Figure 3D). It was not possible to establish if CR_dMFD was recruited to SGs due to its high aggregation propensity. These results indicate that CREST recruitment to SGs is restricted by its limited occurrence in the cytoplasm and that the presence of C-terminal domain (CT, aa. 317-396) and MFD is necessary and sufficient for SG targeting of CREST.

Cytoplasmic aggregates of full-length CREST-GFP observed in a fraction of cells that also display large nuclear aggregates (similar to illustrated in Figure 1A) were negative for SG or P-body markers under basal conditions or after stress, although in stressed cells these aggregates were found in the vicinity of SGs (Figure 3E,F). Large cytoplasmic aggregates formed by CR_dMFD protein also did not contain core SG proteins even in stressed cells (Figure 3G).

### CREST aggregation affects paraspeckles

**Figure 4:**
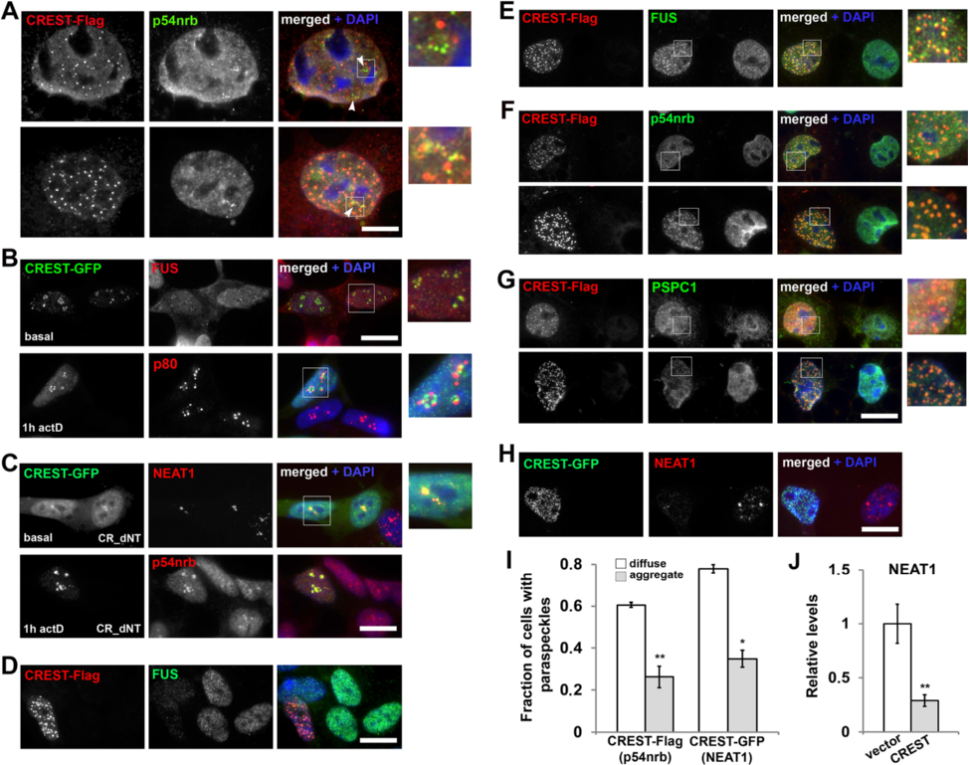
**CREST aggregation disrupts paraspeckles. (A)** CREST aggregates might originate from the sites of paraspeckle formation. Paraspeckles (anti-NONO/p54nrb staining, arrowheads) and CREST nuclear aggregates exist as distinct structures in COS7 cells (top panel). In a fraction of cells CREST aggregates are found in close apposition to/partially overlapping with paraspeckles (bottom panel). **(B)** In response to transcriptional inhibition CREST redistributes to the same nucleolar caps as a typical paraspeckle protein FUS but not to the caps formed by coilin p80. **(C)** CREST lacking autoregulatory domain is efficiently recruited in paraspeckles (top panel) and redistributes to nucleolar caps (bottom panel). **(D)** Endogenous FUS is not essential for nuclear aggregation of CREST. Cells were co-transfected with FUS siRNA and a plasmid to express CREST-Flag and were analysed 48 hours post-transfection. **(E-G)** CREST efficiently sequesters endogenous FUS into dot-like nuclear aggregates in COS7 cells. In contrast, two other paraspeckle components, p54nrb and PSPC1, are not recruited to small and medium-sized CREST aggregates (F and G, top panels), and are detected in aggregates only in nuclei with extensive CREST aggregation (F and G, bottom panels). **(H, I)** Presence of CREST aggregates in the nucleus negatively affects paraspeckles. The fraction of cells with paraspeckles among COS7 cells expressing CREST-Flag (anti-p54nrb staining) or CREST-GFP (FISH with NEAT1 probe) was quantified separately for cells with diffuse CREST distribution and with nuclear CREST aggregates (mean ± s.e.m, *p < 0.05, **p < 0.01; 150-250 cells counted from each of the four or three independent experiments). **(J)** NEAT1 levels are decreased in CREST-expressing cells. Untagged CREST or GFP (vector) were expressed in SH-SY5Y cells for 24 hours; NEAT1 levels were measured by qPCR (**p < 0.01; results from four independent experiments run in duplicates). Scale bars, A – 5 μm; B-G – 10 μm.

Dysfunction of the paraspeckle has been recently implicated in pathogenesis of ALS [18,19]. Although the role of CREST in this nuclear body is not clear, it is unlikely to be essential for paraspeckle assembly because the majority of stable cell lines display normal paraspeckles despite very low levels of CREST expression. To further examine the involvement of CREST in these nuclear bodies, we used COS7 cells, which possess prominent paraspeckles. In the majority of cells displaying nuclear dot-like aggregates of CREST-Flag, these structures were clearly distinct from paraspeckles visualized by staining for a core paraspeckle protein NONO/p54nrb (Figure 4A, top panel). However, careful examination revealed that a fraction of CREST aggregates was adjacent to paraspeckles (Figure 4A, bottom panel). Consistently, similar to paraspeckles, these aggregates are often seen on the border of speckles (Figure 1C). Therefore, sites of paraspeckle assembly might serve as sites of nucleation of CREST aggregates that radiate from paraspeckles and subsequently become scattered in the nucleoplasm. CREST behaves as a typical paraspeckle protein upon transcriptional repression, i.e. becomes recruited to nucleolar caps (Figure 2A) and Ref. [14]). These nucleolar caps match those formed by other paraspeckle proteins such as FUS, and are distinct from nucleolar caps formed by coilin p80 (Figure 4B). Furthermore, CREST deletion variants lacking an autoregulatory domain, CR_dNT and CR_dNT-Met, unlike other deletion mutants, are highly enriched in paraspeckles regardless expression levels and can be readily recruited to actinomycin D-induced nucleolar caps (Figure 4C and data not shown).

We next asked if CREST is able to recruit other paraspeckle proteins into its nuclear aggregates. Indeed, a core paraspeckle component, FUS, was efficiently sequestered into virtually all nuclear aggregates formed by CREST-Flag or CREST-GFP regardless their size and abundance (Figure 4E, Additional file 3: Figure S2B). However, FUS is not essential for CREST aggregation, since siRNA-mediated FUS knockdown did not affect nuclear aggregate formation by CREST-Flag (Figure 4D). Other major paraspeckle proteins, NONO/p54nrb and PSPC1, were not detected in small CREST aggregates (Figure 4F,G, top panels) but co-aggregated with CREST in those nuclei where most of CREST pool was present in the form of larger aggregates (Figure 4F,G, bottom panels). These observations suggest that FUS recruitment to CREST aggregates is highly specific while NONO/p54nrb and PSPC1 can be non-specifically trapped in these aggregates upon their growth. Since FUS is essential for paraspeckle integrity and contributes to the maintenance of NEAT1 levels [14,19], its entrapment in aggregates can negatively affect paraspeckles, including via NEAT1 downregulation. Indeed, COS7 cells expressing CREST-Flag and developing nuclear aggregates, contained paraspeckles (visualised with anti-NONO/p54nrb staining) less frequently than cells with diffuse protein only (Figure 4I). Similarly, among CREST-GFP expressing COS7 cells significantly fewer aggregate-containing cells possessed NEAT1-positive paraspeckles compared to non-transfected cells or cells with diffuse CREST distribution (Figure 4H,I). Furthermore, when we measured NEAT1 levels in neuroblastoma cells expressing untagged CREST or GFP vector, we observed a significant decrease in the transcript abundance in CREST-expressing cells (Figure 4J).

### CREST co-aggregates with FUS but not ALS-associated proteins TDP-43 or TAF15

**Figure 5:**
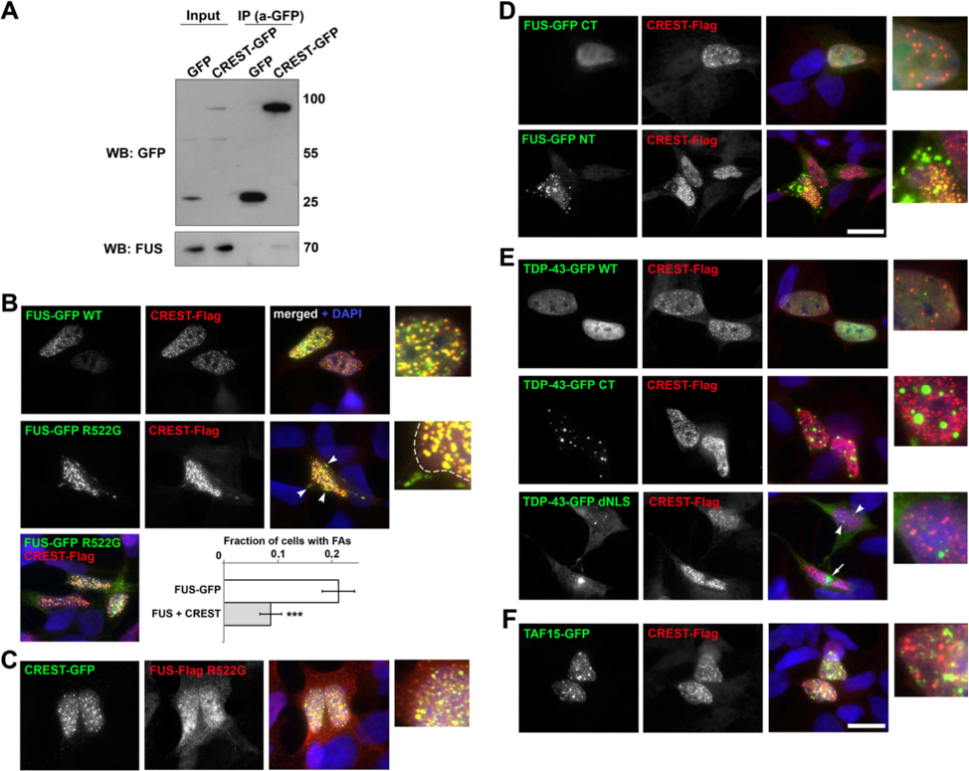
**CREST co-aggregates with FUS but not with TDP-43 or TAF15. (A)** Endogenous FUS co-immunoprecipitates with CREST-GFP. Pull-down of GFP-tagged CREST from transfected cells was performed with GFP-Trap beads as described in Material and methods, endogenous FUS was detected in the immunoprecipitate (IP) by Western blotting (WB). **(B)** CREST-Flag recruits GFP-tagged FUS (top panel) and its cytoplasmically mislocalised variant bearing R522 substitution (FUS R522G, middle panel) into nuclear aggregates upon co-expression in SH-SY5Y cells. In a small fraction of co-expressing cells the latter variant also co-aggregates with CREST in the cytoplasm (middle, arrowheads, border of the nucleus is indicated by a dashed line in the inset) but in the majority of such cells it is trapped in the nucleus leading to its lowered cytoplasmic levels and significant decrease in the proportion of cells bearing cytoplasmic FUS aggregates (FAs, bottom panel). The number of cells with aggregates was quantified for cells expressing GFP-tagged FUS R522G only (FUS-GFP) and those co-expressing GFP-tagged FUS R522G and CREST-Flag (FUS + CREST). The bar chart shows the fraction of cells (mean ± s.e.m.) bearing FAs (***p < 0.001; at least 100 cells counted per variant from three independent experiments). **(C)** CREST-GFP and FUS-Flag with R522G substitution co-aggregate. **(D)** N-terminally truncated protein (FUS-GFP CT, aa. 360-526) cannot be recruited to CREST-Flag aggregates (top panel), while C-terminally truncated FUS (FUS-GFP NT, aa.1-359) retains the ability to co-aggregate with CREST (bottom panel). **(E)** CREST does not sequester wild-type TDP-43 into nuclear aggregates and is not recruited to cytoplasmic (arrow) or nuclear (arrowheads) aggregates formed by mislocalised TDP-43 (TDP-43-GFP dNLS) or C-terminal TDP-43 fragment (TDP-43-GFP CT, aa.193-414). NLS of TDP-43 was deleted to achieve cytoplasmic re-distribution and aggregation of the protein. **(F)** Nuclear aggregates of Flag-tagged CREST and GFP-tagged TAF15 do not overlap. Scale bars, 10 μm.

Previously reported results of co-immunoprecipitation experiments demonstrated that two ALS-associated proteins, CREST and FUS, interact in vivo [4]. However, we identified a significant cross-reactivity of the anti-CREST antibody used in the above study with FUS protein (Additional file 4: Figure S3). Nevertheless, we also found that endogenous FUS is efficiently recruited into nuclear CREST aggregates (Figure 4E). To confirm CREST-FUS interaction in vivo, we transfected cells with a construct to express CREST-GFP and performed immunoprecipitation using GFP-Trap beads. Endogenous FUS was co-immunoprecipitated with CREST in this cellular system indicating that the proteins indeed interact in vivo (Figure 5A).

We went on to study FUS-CREST co-aggregation in more detail and establish if it is specific for FUS. Co-expression of CREST-Flag and FUS-GFP led to their robust co-aggregation in the nucleus (Figure 5B, top panel), seemingly, via sequestration of FUS into dot-like CREST aggregates. This was also true for cytoplasmically mislocalised FUS mutant bearing a substitution in its nuclear localisation signal, FUS R522G (Figure 5B, middle panel). Although R522G mutation leads to dramatic shift to the cytoplasm and subsequent cytoplasmic aggregation of FUS [22,23], when co-expressed with CREST-Flag, FUS-GFP R522G was present in the cytoplasm at low levels and the percent of cells bearing cytoplasmic FUS aggregates was significantly decreased (Figure 5B, bottom panel). We concluded that aggregating CREST-Flag is able to recruit and retain FUS in the nucleus leading to its lowered cytoplasmic levels and interfering with its cytoplasmic aggregation. Interestingly, cells that formed cytoplasmic FUS R522G aggregates also contained overexpressed CREST within those aggregates (Figure 5, middle panel, arrowheads). Similarly, CREST-GFP co-aggregated with FUS-Flag R522G (Figure 5C). The region responsible for co-aggregation of FUS with CREST lies in the N-terminal part of the FUS molecule, since a variant lacking all domains downstream of the RRM (NT-RRM, aa. 1-359) was still able to co-aggregate with CREST, but no recruitment into CREST aggregates was observed for the C-terminal FUS fragment (CT, aa.360-526) (Figure 5D). In contrast, neither normal TDP-43, nor TDP-43 rendered cytoplasmiс by deletion of its NLS, nor C-terminal TDP-43 fragment (aa.193-414), showed co-aggregation with CREST (Figure 5E). Furthermore, aggregates formed in the nucleus by another ALS-associated protein structurally highly similar to FUS, TAF15, were clearly distinct from nuclear CREST aggregates (Figure 5F).

### Q388stop mutation alters steady-state levels and aggregation propensity of CREST protein

**Figure 6:**
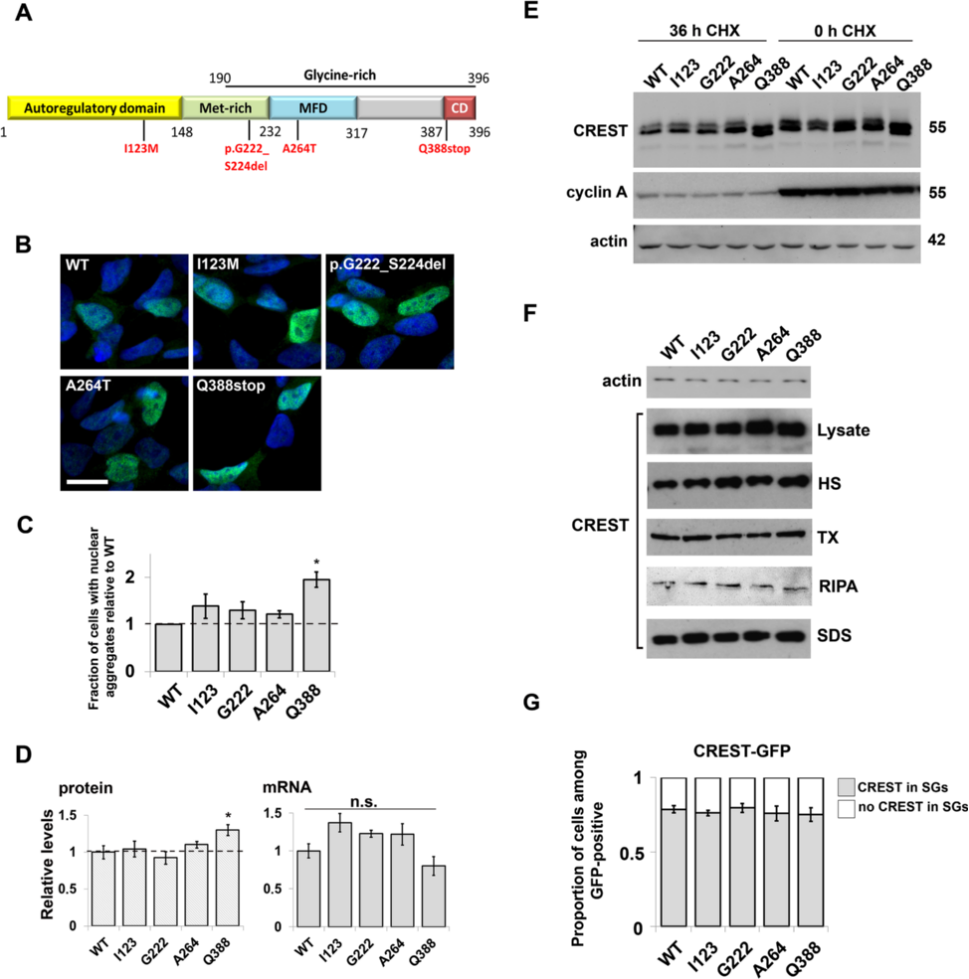
**ALS-associated CREST mutation Q388stop increases steady-state levels of the protein and its ability to aggregate. (A)** A map of CREST with the positions of single amino acid substitutions or a deletion found in ALS indicated. **(B)** All CREST variants are localized predominantly to the nucleus in SH-SY5Y cells. **(C)** Q388stop mutant aggregates in the nucleus more often compared to the normal protein and other mutants. Among cells expressing each of the untagged CREST variants those with aggregates in the nucleus were quantified on the entire coverslip 24 hours post-transfection and obtained values were normalized against the value for wild-type CREST. The bar chart shows means ± s.e.m. of these normalized values from four independent experiments (*p < 0.05). **(D)** Steady-state protein levels for Q388stop mutant variant are increased compared to the wild-type protein without significant difference at the transcript level. The bar charts show means ± s.e.m. of seven independent experiments (*p < 0.05, Mann-Whitney U-test). **(E)** All CREST variants are stable proteins with half-lives of approximately 36 hours. Cycloheximide was used to block protein synthesis; levels of a short-lived protein, cyclin A, were measured to confirm successful block of translation. **(F)** Solubility of mutant CREST variants upon sequential protein extraction was comparable to that of wild-type protein. Fractionation was performed as described in Materials and methods. **(G)** CREST mutants do not affect the ability of the protein to be recruited to stress granules. The fraction of cells with CREST-positive stress granules after sodium arsenite exposure was quantified in the population of CREST-GFP expressing cells for each variant (at least 100 cells counted per variant in each of the two independent experiments). Scale bar, 10 μm.

Recently four mutations were identified in CREST in ALS patients [4,5]. However, the basis of their pathogenicity is still not clear. The mutations do not cluster but are spread along the molecule, each found in a different domain (Figure 6A). We created constructs for the expression of these four variants as untagged proteins. The proteins displayed predominantly nuclear distribution indistinguishable from the wild-type protein (Figure 6B). All variants occasionally formed nuclear dot-like aggregates similar to those shown in Figure 1A for untagged wild-type CREST; the frequency of cells bearing such aggregates was comparable between the variants except Q388stop, a mutant lacking eight amino acids at its C-terminus. The latter aggregated in the nucleus significantly more often than wild-type protein or any other studied mutant (p = 0.0169; Figure 6C). All CREST variants were expressed in cell lines studied at similar levels, however, densitometric analysis revealed that steady-state levels of Q388stop variant were elevated (p = 0.0483) compared to other variants despite insignificant differences at mRNA level (Figure 6D). To assess if CREST mutations alter protein stability, we estimated the proteins’ half-lives using a pulse chase with cycloheximide. Overexpressed CREST was stable, with half-life of approximately 36 hours, without significant differences between the variants but again with the exception of Q388stop, which displayed increased stability (Figure 6E). The propensity of CREST mutants to aggregate was estimated using the sequential extraction protocol described above. All variants behaved very similarly to the non-mutated CREST without marked changes in the abundance in different fractions (Figure 6F). We did not observe any differences between the mutants in their ability to be recruited to SGs or paraspeckles, or co-aggregate with FUS protein (Figure 6G, Additional file 3: Figure S2).

### CREST overexpression negatively affects dendritic complexity in cultured hippocampal neurons and causes retina degeneration in transgenic Drosophila

**Figure 7:**
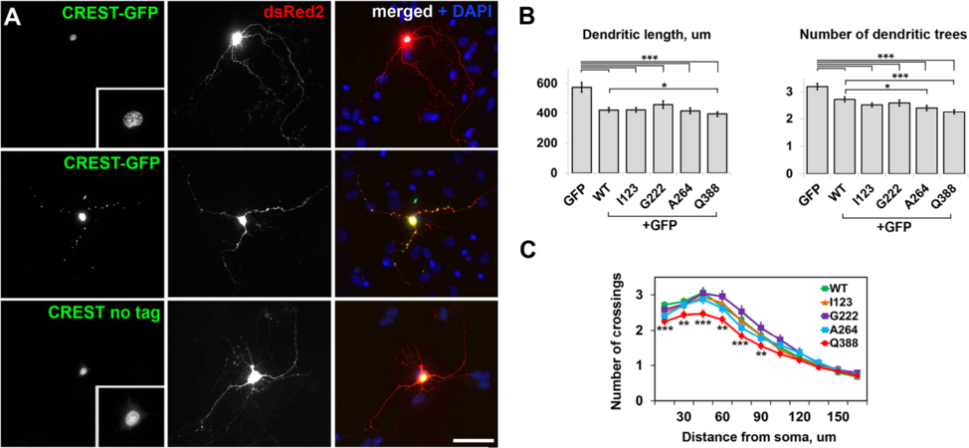
**Overexpression of normal or mutant CREST affects complexity of dendritic tree in primary hippocampal neurons. (A)** CREST-GFP is largely confined in the nucleus when expressed in primary mouse neurons but is also found in multiple dot-like aggregates in the processes in some cells. Untagged CREST displays nuclear distribution in neurons. CREST-GFP or untagged CREST was co-transfected into primary hippocampal neurons together with dsRed2 to visualise neuronal morphology and allowed to express for 48 hours prior to analysis. **(B)** Total dendritic length in micrometers measured in mouse hippocampal neurons expressing CREST variants. Mouse primary hippocampal neurons were co-transfected with vectors to express GFP (to visualize neuronal morphology) and each of the untagged CREST variants and allowed to express the proteins for 48 hours prior to analysis. Control neurons were transfected with GFP-expressing vector only. The bar charts show means ± s.e.m. for at least 100 neurons per variant from four independent experiments (*p < 0.05, ***p < 0.001). **(C)** Sholl analysis of the same neurons as in B. Number of dendrite intersections of 15-μm spaced shells as a function of the radial distance from the soma was plotted. **p < 0.01, ***p < 0.001. Scale bar, 50 μm.

In a previous study an inhibitory effect of mutant CREST variants Q388stop and I123M on KCl-induced neurite outgrowth in cultured mouse cortical neurons has been reported [4]. Because CREST is prone to aggregation and sequesters some important proteins into aggregates, it is feasible that its overexpression per se might be deleterious to neurons. We therefore examined the effect of normal CREST and its mutants on the complexity of dendritic tree in unstimulated primary mouse hippocampal neurons. In these cells both GFP-tagged and untagged CREST displayed similar distribution with mainly nuclear localization, and frequent aggregation in the nucleus and occasionally in the processes (Figure 7A); aggregation increased in a concentration-dependent manner. Untagged CREST was used for further analysis due to its more physiological nature. Hippocampal neurons isolated from mice on postnatal day 3 and cultured for 5 days were co-transfected with a vector for GFP expression to visualise neuronal morphology, and each of the CREST variants. The cells were allowed to express the proteins for 48 hours (see Methods for details). Total dendritic length was significantly decreased in CREST-expressing neurons compared to neurons transfected with vector only and was comparable between the variants (Figure 7B). However, this parameter was lower (p = 0.049983) for Q388stop variant compared to wild-type CREST (Figure 7B). Similarly, CREST-expressing cells exhibited fewer dendritic trees, and for Q388stop and to a lesser extent A264T, this effect was even more pronounced than for wild-type protein (Figure 7B). Sholl analysis of CREST expressing neurons also revealed a significant difference only for the Q388stop mutant: the number of shell intersections for neurons expressing this protein was significantly lower compared to that for wild-type protein (Figure 7C).

**Figure 8:**
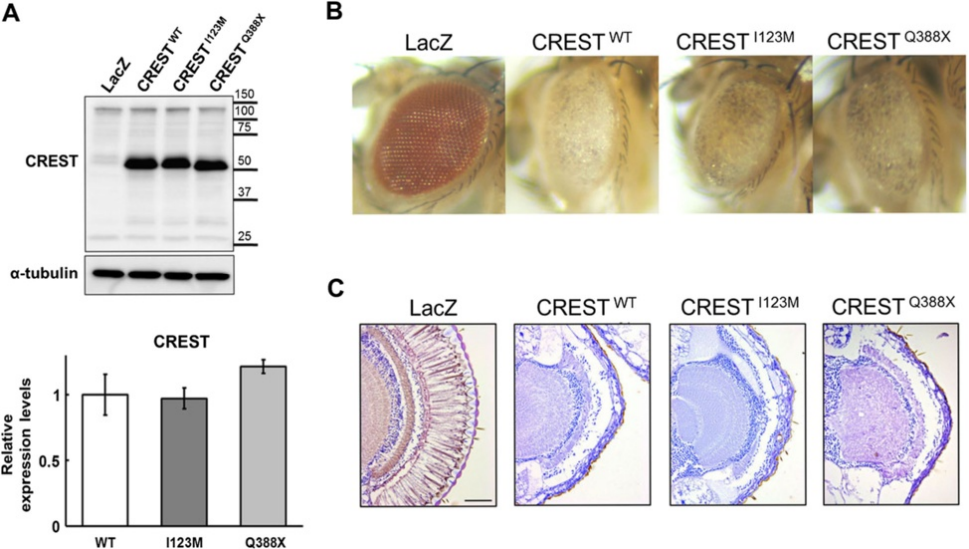
**Overexpression of normal or mutant CREST induces retinal degeneration in** ***Drosophila melanogaster.*** **(A)** Protein expression levels of CREST variants in the heads of transgenic flies of each line as determined by Western blotting and subsequent quantification of band intensities (mean ± s.d). **(B)** Images of external head surface of 5-day-old flies overexpressing wild-type CREST, CREST I123M, CREST Q388stop or lacZ in the retina. Overexpression of wild-type CREST or its variants in photoreceptor neurons results in rough and depigmented eyes. **(C)** H&E staining of retinal sections of 5-day-old flies overexpressing wild-type CREST or its variants reveals disruption of regularly ordered arrays of photoreceptor neurons. Data for representative transgenic fly lines with similar (intermediate) level of CREST protein expression are shown (#7, #7 and #2 for WT, I123M and Q388stop variants, respectively, – see Additional file 5: Figure S4). Scale bar, 50 μm.

To address possible effects of elevated CREST levels in vivo, we generated Drosophila melanogaster lines overexpressing untagged normal or mutant human protein using GAL4-UAS system (Additional file 5: Figure S4, Figure 8A). UAS-CREST wild-type, I123M and Q388stop transgenic lines were crossed with gmr-GAL4 line to drive the expression of proteins in the retinal photoreceptor neurons. External morphology of the heads of 5-day-old flies overexpressing these variants was characterized by rough and de-pigmented eyes compared to those overexpressing lacZ as a control protein, without obvious differences between the lines (Figure 8B). Histological analysis revealed marked disruption of the regularly ordered arrays of photoreceptor neurons with thinned retina (Figure 8C). Thus, overexpression of either normal or mutant CREST in photoreceptor neurons of a fly is sufficient to induce severe degeneration.

## Discussion

Our study presents evidence that CREST possesses three properties common to several proteins associated with ALS: i) high propensity to aggregate in cells; ii) recruitment into stress granules; and iii) ability to modulate paraspeckle integrity. There is a growing body of evidence that protein aggregation and altered function of SGs (and likely, paraspeckles) are important factors in ALS pathogenesis, and our observations also point to these processes as potential culprits for the development of pathological changes in ALS cases with CREST mutations.

In the previous studies devoted to cellular localization and functions of CREST it has been proposed that nuclear structures formed by CREST represent a type of nuclear body that recruits CBP protein and thereby regulates its functions [13]. Here we show that CREST structures appearing upon protein accumulation in the nucleus correspond to irreversibly aggregated entities formed due to the high aggregation propensity of CREST, since they grow in time- and concentration-dependent manner and do not dissipate in response to transcriptional repression like genuine nuclear bodies, paraspeckles and Cajal bodies [24,25]. In line with this, CREST accumulates in detergent-insoluble fractions when overexpressed in cultured cells. It cannot be ruled out that at early stages of their formation, nuclear CREST aggregates represent physiologically relevant and functional foci recruiting factors such as CBP to certain genome locations. However, in certain conditions CREST in these foci may start aggregating uncontrollably thereby transforming them into pathological aggregates. This can be caused by abnormally high accumulation of CREST in the nucleus and, consequently, in the foci, or by lower concentration threshold of uncontrollable aggregation for mutated CREST. A similar scenario has been suggested for transformation of cytoplasmic RNA granules enriched in proteins with prion-like domains into proteinaceous inclusions in proteinopathies [22,23]. CREST also has a prion-like domain in its structure, and the latter was proposed to play a role both in physiological and pathological aggregation [15-17]. Since at present the nature of these CREST structures is unclear, in this study they are collectively referred as “aggregates” regardless their physiological significance. It should be kept in mind that accumulation of very high levels of the protein observed in some cells upon its overexpression is unlikely to be achieved for the endogenous protein. However, in our study nuclear CREST aggregates started appearing already in cells with low levels of exogenous protein indicating a low threshold for its multimerization. Further studies using more physiological models, such as neurons differentiated from iPS cells of patients with CREST mutations or cells with modified CREST gene, are required to establish the molecular nature of CREST aggregates at different stages of their formation as well as functional relevance of CREST multimerization.

It has been shown previously that the N-terminal autoregulatory domain of CREST is largely dispensable for its localization to nuclear aggregates [13] however we also noted that the CR_dNT mutant lacking this domain has diminished aggregation propensity. Interestingly, unlike similar domains of FUS and TDP-43 [26-28], the C-terminal transactivation domain of CREST cannot aggregate in isolation despite its prion-like properties. Thus, the aggregation propensity of CREST is defined by cooperative action of its C-terminal and N-terminal domains, while MFD may limit it. Importantly, in our study only CREST deletion variants with preserved ability to aggregate were capable of SG and paraspeckle recruitment. This correlation provides further support for the notion that the ability of proteins bearing a prion-like domain to aggregate reversibly (i.e. “physiological” aggregation) is prerequisite for their entry into RNA granules such as SGs and paraspeckles [15-17]. Seemingly, such proteins have to be tightly regulated, and modulation of CREST aggregation propensity by autoregulatory domain and MFD is an example of such regulation. Even subtle changes in the structure of such domains may lead to uncontrollable aggregation and downstream events deleterious for neurons. We also demonstrated that both autoregulatory domain and MFD of CREST contribute to nuclear localization of the protein. Impairment of these domains’ function may therefore result in cytoplasmic redistribution of the protein, similar to redistribution of FUS bearing ALS-linked mutations in its nuclear localization signal [22,23].

We also showed that nuclear aggregates of CREST are able to trap certain proteins such as FUS, which could lead to loss of their function. Therefore, changes in CREST levels (e.g. due to increased stability) or its solubility, triggered by any external or internal factor including mutation, could initiate aggregation of the protein in the nervous system, which in turn would affect CREST binding partners important for neuronal homeostasis. There are several reports of increased stability [29-31] and/or higher aggregation propensity [28,32,33] of ALS-associated TDP-43 mutants compared to non-mutated protein. Similarly, we have demonstrated that at least one of the ALS-linked mutations in CREST, Q388stop, affects protein stability and renders it more aggregate-prone. An opposite situation, where CREST would be sequestered into aggregates formed by other proteins, particularly FUS, is also possible. Our attempts to establish if CREST pathology is present in FUSopathies were precluded by cross-reactivity of the anti-CREST antibody, suitable for histological staining, with FUS. When a truly specific antibody becomes available it would be interesting to determine the extent of CREST involvement in histopathology typical of FUSopathies.

Altered metabolism of SGs is now widely acknowledged to be contributory to ALS pathogenesis [3,34-38], and recently dysfunction of another RNA granule, the paraspeckle, has emerged as a possible factor in ALS pathology. Paraspeckles are built on a long non-coding RNA NEAT1 and recruit a subset of proteins, including FUS and to a lesser extent TDP-43 [14]. Functionally, this RNA granule is involved in the nuclear retention of specific RNAs [39], regulation of gene expression and possibly the cellular stress response [40,41]. Results of several studies suggest a role for paraspeckle proteins and NEAT1 in ALS/FTLD disease pathogenesis [42-49]. Furthermore, it has been specifically demonstrated that paraspeckle assembly triggered by upregulated NEAT1 synthesis occurs in motor neurons at the early stage of ALS development, suggesting a protective role against various insults, which might be particularly important for motor neuron welfare [18]. We have also established links between loss and gain of function of FUS protein and disrupted paraspeckle assembly in ALS-FUS [19]. This allowed us to propose a model whereby aggregation of FUS in the cytoplasm leads to its nuclear loss accompanied by depletion of other paraspeckle proteins, which in turn may disable protective paraspeckle assembly and contribute to neurodegeneration in FUSopathies. A similar scenario may occur in ALS cases caused by CREST mutations, where aggregating protein can sequester FUS (and perhaps other paraspeckle proteins) into aggregates and attenuate protective paraspeckle formation.

We observed no overt phenotypes specific to identified ALS-associated CREST mutations except for Q388stop. Instead, we showed that excess of CREST affects neuron morphology independently of the presence of mutations. Moreover, in transgenic fly models, all studied protein variants, including non-mutated protein, were highly toxic and induced severe retinal degeneration. Therefore, alterations of CREST stability and consequently, intracellular protein level, which might occur as a result of mutation or triggered by external factors, would be deleterious for neurons, the only type of cells expressing high levels of CREST. Q388stop mutation was predicted to be highly damaging since it affects the prion-like domain and removes a CBP-interaction motif of CREST [4]. We showed that this mutation also increases steady-state protein levels, promotes CREST aggregation and perhaps as a result, negatively affects branching and outgrowth of neuritis in cultured neurons. No gross differences in subcellular localization, aggregation capacity, stability, SG/paraspeckle recruitment or in vivo toxicity of other mutants compared to wild-type protein were registered indicating the need for fine structural and functional assays to further probe their pathogenicity. The fact that alterations in the structure and properties of CREST are not immediately recognizable is not surprising as a similar trend was revealed in the studies of mutant variants of other ALS-associated proteins. For example, only some ALS-causative FUS variants with mutations in NLS are redistributed to the cytoplasm in cultured cells, whereas others do not visibly affect nuclear import [50,51]. Similarly, prion-like and glycine-rich domains of TDP-43 are the hot-spots of mutations, but functional consequences of different mutations in these domains are highly variable [51,52]. Studies on post-mortem tissue of patients bearing mutations in CREST would be highly informative for establishing if CREST is indeed another protein whose aggregation leads to neurodegeneration. CREST is essential for the nervous system homeostasis as evidenced by the severe neurological phenotype of CREST deficient mice [6] as well as its high expression levels specifically in postmitotic neurons [9]. Therefore, it is highly likely that mutations identified in ALS only slightly compromise the protein structure allowing normal development of the nervous system, but nevertheless are sufficient to trigger a severe neurodegenerative disorder in the adulthood. Our studies in vitro and in vivo suggested that altered protein stability and hence its increased levels may be behind this effect. Interestingly, one ALS patient with CREST mutation also carried an amino acid substitution in OPTN gene [5]. It is plausible that in this case the presence of both mutations was necessary for manifestation of the clinical phenotype, providing another example in support of the emerging concept of ALS as an oligogenic disease [53].

In summary, our study provides experimental evidence that CREST can be considered another member of the growing group of ALS-linked aggregation-prone proteins capable of recruitment to SGs and other RNA granules. Until very recently, members of this group were exclusively RNA-binding proteins. However, together with the report on profilin1 recruitment to SGs [54], our study links ALS aetiology with dysfunction of RNA granule components other than RNA-binding proteins.

## Methods

### Expression plasmids, stable cell lines, transfection and treatments

DNA fragments encoding full-length CREST, FUS, TDP-43 and TAF15 and their deletion and mutant variants were produced by RT-PCR amplification with SuperScript III and AccuPrime polymerases (Invitrogen, Life Technologies) from human (SH-SY5Y cells or fetal brain) total RNA using respective primers and cloned into pCR-BluntII-TOPO vector (Invitrogen, Life Technologies). After sequence validation the fragments were subcloned into the pEGFP-C1 vector (Clontech) downstream and in-frame with GFP to produce proteins bearing N-terminal GFP tag; into the pEGFP-N1 vector (Clontech) upstream and separated by a stop codon from GFP to obtain non-tagged proteins; and into the pCMV4-Flag vector (Sigma) for the expression of Flag-tagged wild-type CREST. SH-SY5Y, HEK293 and COS7 cells were maintained in Dulbecco modified Eagle medium supplemented with 10% foetal bovine serum (Invitrogen, Life Technologies). For immunofluorescence cells were grown on poly-L-lysine coated coverslips. Cells were transfected with expression plasmids or mixture of a plasmid and FUS siRNAs using Lipofectamine2000 (Invitrogen, Life Technologies) according to the manufacturer’s instructions. For plasmid co-transfections equal amounts of plasmid DNA were taken into the reaction. For FUS knockdown FUS-specific SiGENOME SMART pool (M-009497-02, Thermo Scientific) was used. For nucleolus staining living cells were exposed to 10 μg/ml of ethidium bromide for 1 hour prior to fixation. To block transcription, cells were treated with 5 μg/ml actinomycin D (Calbiochem, Merck Millipore) for 1 hour. To induce formation of stress granules, cells were subjected to 5 mM sodium arsenite (Sigma) for 1 hour, 2 μM thapsigargin (Sigma) for 4 hours or 50 μM 15-deoxy-delta 12,14-prostaglandin J2 (15d-PGJ2, Cayman Chemical) for 1 hour.

### Immunofluorescence on coverslips

Cells were fixed with 4% paraformaldehyde on ice for 15 min, followed by washes with PBS and 5 min permebealization in cold methanol. After three washes with PBS and blocking in 5% goat serum/PBS/0.1% Triton-X100 for 1 h at room temperature coverslips were incubated with primary antibodies diluted in blocking solution for 1 h at room temperature or at 4°C overnight. Alexa Fluor-conjugated anti-mouse or anti-rabbit immunoglobulins (Molecular Probes, Life Technologies) were used as secondary antibodies (1:1000 in PBS/0.1% Triton-X100) and cell nuclei were visualized with DAPI. For RNA-FISH, a commercially available NEAT1 and MALAT1 probes (Stellaris® FISH Probes against human NEAT1 5′ segment and human MALAT1, Biosearch Technologies) was used according to the protocol provided by the manufacturer. Fluorescent images were taken using BX61 microscope (Olympus) and processed using CellF software (Olympus). For assessing cytoplasmic redistribution of CREST under stress, fluorescence intensity was measured 24 hours after transfection in naïve and sodium-arsenite treated cells in three 2.5 × 2.5 μm squares randomly chosen in the cytoplasm of a transfected cell using free-access ImageJ software. To estimate the enrichment of full-length CREST and CR_dNT in SGs, fluorescence intensity was measured in three 0.5 × 0.5 μm squares in the cytoplasm free from SGs and in three 0.5 × 0.5 μm squares within SGs of a transfected cell. Mean intensities per cell were calculated and used to calculate standard deviation and standard error of the mean (s.e.m.), as well as ratio SG/cytoplasm.

### Live cell imaging

Time-lapse images were obtained using Leica TCS SP2 MP confocal microscope, equipped with an on-scope incubator with temperature control. SH-SY5Y cells were plated on glass-bottomed dishes (Mattek) and transfected with a plasmid to express CREST-GFP. Before imaging, regular culture media was replaced with HEPES-buffered media (10 mM HEPES-KOH, pH7.5). Cells were visualized under Fluotar L 63 × 1.4 oil objective. A sequence of images was further transformed into a video clip using Leica Application Suite AF software.

### Primary mouse hippocampal cultures

All reagents used for preparation of hippocampal cultures were purchased from Invitrogen, Life Technologies unless stated otherwise. Hippocampi were dissected from mice at postnatal day 3, digested for 40 minutes in 0.1% trypsin in HBSS supplemented with 10 mM Hepes and 1 mM pyruvate. After mechanical dissociation in Neurobasal A medium containing 50 U/ml penicillin/streptomycin, 0.2% β-mercaptoethanol, 500 μM L-Glutamine and 10% horse serum, hippocampi were centrifuged for 5 minutes at 1,500 rpm. Pellets were resuspended in fresh medium and plated on poly-L-lysine coated coverslips. One day after plating the medium was changed to serum-free medium containing B27. Mixed neuronal-glial cultures were transfected on DIV5 using Lipofectamine2000 according to the standard procedure, except Lipofectamin-DNA complexes which were left for 1 hour and subsequently replaced with normal culture medium. Cells were fixed and stained 48 hours after transfection. Estimation of dendritic length, number of dendritic trees and Sholl analysis were performed using Matlab5 software. At least 100 neurons from four independent experiments were analysed per variant.

### Primary antibodies

Commercially available primary antibodies against the following antigens were used: CREST (rabbit polyclonal, 12439-1-AP, Proteintech); FUS (mouse monoclonal against C-terminus, Santa Cruz, sc-47711; rabbit polyclonal, ab84078, Abcam); p54nrb (rabbit polyclonal C-terminal, Sigma); TIAR (mouse monoclonal, BD Biosciences); G3BP1 (mouse monoclonal, BD Biosciences); Dcp1a (rabbit polyclonal C-terminal, Sigma); anti-Flag M2 (mouse monoclonal, Sigma); PSPC1 (rabbit polyclonal C-terminal, Sigma); GFP (Living Colours® rabbit polyclonal, Clontech, #632593); SMN (mouse monoclonal, BD Biosciences); cyclin A (rabbit polyclonal, Santa Cruz, sc-751); p80 coilin (mouse monoclonal, BD Biosciences); PML (chicken polyclonal, a kind gift from Prof. Ronald Hay, Dundee); phosphorylated tau (mouse monoclonal, clone AT8, Thermo Scientific); beta-actin (mouse monoclonal, clone AC15, Sigma); alpha-tubulin (mouse monoclonal, clone DM1A, Sigma). Primary antibodies were used at 1:1,000 dilution for all applications.

### Protein stability

SH-SY5Y cells were transfected with corresponding constructs and allowed to express the protein for 24 hours. To block translation, cycloheximide (Sigma) was added to a final concentration of 20 μg/ml; cells were harvested after 8, 12, 24, 36 and 48 hours of exposure.

### Sequential protein extraction

HEK293 cells were transfected with corresponding constructs and harvested 24 hours post-transfection. Cells were disrupted mechanically in high-salt buffer containing protease inhibitors and centrifuged at 13,000 rpm to remove cell debris; a small amount was kept as total lysate. Samples were subjected to ultracentrifugation at 48,000 rpm for 20 min and supernatant was recovered as high-salt soluble fraction (HS). Resulting pellets were resuspended in high-salt buffer supplemented with 1% Triton-X100 and centrifuged again under the same conditions. Supernatant was labelled as Triton-X100 soluble fraction (TX). The same steps were repeated with RIPA buffer to obtain RIPA-soluble fraction (RIPA). Final pellets were lysed directly in SDS-PAGE loading buffer to yield SDS-soluble fraction (SDS). Protein amounts in different fractions were normalized against the protein amount in the high-salt fraction. For total lysate (L) and high-salt (HS) fraction 10% of the amount relative to other fractions was loaded.

### RT-PCR and qPCR

Total RNA was isolated using RNeasy mini kit (Qiagen) and possible DNA contamination removed using RNase free DNase kit (Qiagen). First-strand cDNA synthesis was carried out on 500 ng of RNA using SuperScript III reverse transcriptase (Invitrogen, Life Technologies) and random hexamers (Promega) according to manufacturer’s instructions. Quantitative real-time PCR was run in triplicate on an ABI StepOne™ real-time PCR instrument and data were analyzed using StepOne™ Software v2.0 (Applied Biosystems, Life Technologies). cDNA amount for each gene was normalized to that of GAPDH. Primer sequences used were as follows: CREST - forward: 5′- ggttacgcagcaaaccatcc-3′, reverse: 5′-ggatctgctggtactgcgtg -3′; NEAT1 - forward: 5′-cttcctccctttaacttatccattcac-3′, reverse: 5′-ctcttcctccaccattaccaacaatac-3′; GAPDH - forward: 5′-tcgccagccgagcca-3′; reverse: 5′-gagttaaaagcagccctggtg-3′.

### Immunoprecipitation

Cells were washed with PBS, lysed in ice cold IP buffer (PBS/1% Triton-X100) on ice with periodic vortexing for 10 min. Unbroken cells and cell debris were pelleted at 13000 rpm for 15 min, input sample was taken at this point. Cell lysates were incubated with GFP-Trap® agarose beads (ChromoTek) for 2 hours at 4°C. Beads were washed four times with ice cold high-salt buffer (20 mM Tris, 300 mM NaCl, 1% Triton-X100) and bound complexes were eluted from beads by boiling for 5 min at 100°C in SDS-PAGE loading buffer. To remove beads, samples were centrifuged at 13000 × g for 5 min. Samples were then analyzed by Western blotting. For input 10% of final IP sample was loaded.

### Western blotting

For SDS-PAGE loading buffer was used to lyse cells on dishes, followed by denaturation at 100°C for 5 min. After SDS-PAGE, proteins were transferred to PVDF membrane by semi-dry blotting followed by blocking, incubation with primary and HRP-conjugated secondary (GE Healthcare) antibodies and ECL detection as described previously [55]. Equal loading was confirmed by re-probing membranes with antibodies against beta-actin or alpha-tubulin.

### Semi-Denaturating Detergent Agarose Gel Electrophoresis (SDD-AGE)

A protocol described previously [56] was used with modifications indicated below. Briefly, SH-SY5Y cells were harvested 24 hours post transfection in PBS-1% Triton-X100, left on ice for 20 min with periodic vortexing and centrifuged at 17,000 × g. To obtain a positive control for the presence of amyloid aggregates the spinal cord of a 6-month old transgenic TauP301S mouse was processed in parallel with cell lysates. Supernatants were mixed with equal amounts of 2X SDD-AGE loading buffer (1XTAE, 5% glycerol and 1% SDS) and run in 1.5% agarose containing 0.1% SDS. Proteins from the gel were transferred to nitrocellulose membrane using capillary transfer and the membrane was subjected to Western blotting using anti-CREST and anti-phosphorylated tau antibodies as described above.

### Generation and characterization of transgenic flies

Constructs encoding human wild-type or mutant CREST, or lacZ in pUAST vector, were injected into w1118 embryos to produce transgenic flies as described previously [57,58]. At least three independent transformant lines were analyzed per construct. gmr-GAL4 and UAS-lacZ lines were obtained from the Bloomington Drosophila stock center. For immunoblot analysis, heads of 5-day-old flies were dissected and lysed in Laemmli sample buffer for SDS-PAGE containing 2% SDS. For external surface observation, 5-day-old flies were anesthetized with CO2 and observed with zoom stereo microscopy (Olympus SZ-PT). For histochemical analyses, heads of 5-day-old adult transgenic flies were dissected, collected, briefly washed in phosphate buffered saline (PBS), and fixed with 4% paraformaldehyde containing 0.1% Triton X-100 at room temperature for 2 hours. After brief wash in PBS, tissues were dehydrated by graded ethanol, cleared in butanol and embedded in paraffin. Four-micrometer thick coronal sections were stained with hematoxylin and eosin (H&E) [58].

### Statistics

Statistical analysis was performed with Mann-Whitney *U*-test using STATISTICA 6.0 software.

## Additional files

Additional file 1: Figure S1. Overexpressed CREST aggregates in a dose-dependent manner in COS7 cells. Flag- or GFP-tagged CREST protein displays diffuse and fine-granular distribution in the nucleus of low-expressing COS7 cells and forms dot-like aggregates as it accumulates. In cells with high levels of the protein, large nuclear aggregates together with cytoplasmic accumulation/aggregation are observed. Cells were analysed 24 hours post-transfection. Scale bar, 10 μm. Additional file 2: Video S1. Time- and concentration-dependent aggregation of CREST in the nucleus. SH-SY5Y cells were transfected with the expression plasmid encoding CREST fused to GFP and imaged 8 hours post-transfection. Note the fusion of dot-like CREST aggregates to each other and aggregate growth with time. QuickTime Player file (.mov). Additional file 3: Figure S2. ALS-linked CREST mutations do not alter the protein’s ability for stress granule or paraspeckle recruitment. (A) All CREST variants are recruited to sodium arsenite induced stress granules. (B) All CREST mutants sequester endogenous FUS protein into nuclear aggregates. (C) Mutations in CREST do not affect its enrichment in paraspeckles in low-expressing SH-SY5Y cells. Scale bars, A, B - 10 μm, C - 5 μm. Additional file 4: Figure S3. Polyclonal anti-CREST antibody is cross-reactive to human FUS protein. (A) Aggregates formed by FUS-GFP R522G or FUS deletion mutant NT-RRM lacking C-terminal domains displayed high immunoreactivity with CREST antibody. Aggregates of GFP-tagged TDP-43 with deleted NLS were not recognised by this antibody ruling out possible cross-reactivity with GFP tag or non-specific recognition of aggregated protein species. (B) Full-length FUS and its deletion mutants are recognized by CREST antibody on Western blots. Specific bands corresponding to GFP-tagged FUS and its deletion mutants dRRM (lacking RRM), dR-R (lacking RRM and RGG3) and NT-RRM (lacking entire C-terminus, amino acids 360-526) are indicated with asterisks. Open arrow points to endogenous CREST protein and black arrows point to non-specific bands. Scale bar, 10 μm. Additional file 5: Figure S4. CREST overexpression in *Drosophila melanogaster* retinal neurons results in retinal degeneration. (A) Transgenic fly lines expressing normal human CREST or its mutants I123M and Q388stop in retinal photoreceptor neurons with different levels of protein expression were generated. Protein levels were measured in the heads of transgenic flies by Western blotting using anti-CREST antibody and band intensities were quantified (bar chart shows mean ± s.d., CREST WT line #2 = 1.0). (B,C) Expression of wild-type or mutant CREST leads to severe retinal degeneration as evidenced by eye depigmentation (B) and its abnormal histology (C, H&E staining). The chromosome with transgene insertion (Ch) for each line is indicated.

## Abbreviations

15d-PGJ2: 15-deoxy-delta 12, 14-prostaglandin J2
ALS: Amyotrophic lateral sclerosis
FTLD: Frontotemporal lobar degeneration
MFD: Multifunctional domain
NEAT1: Nuclear enriched abundant transcript 1
RNP: Ribonucleoprotein
RRM: RNA recognition motif
SA: Sodium arsenite
SDD-AGE: Semidenaturating detergent agarose gel electrophoresis
SG: Stress granule
