# Supplementary figures and images for: Calcium-responsive transactivator (CREST) protein shares a set of structural and functional traits with other proteins associated with amyotrophic lateral sclerosis
Source: Mol Neurodegener. 2015 Apr 10;10:20. doi: 10.1186/s13024-015-0014-y (PMC4428507; doi:10.1186/s13024-015-0014-y)

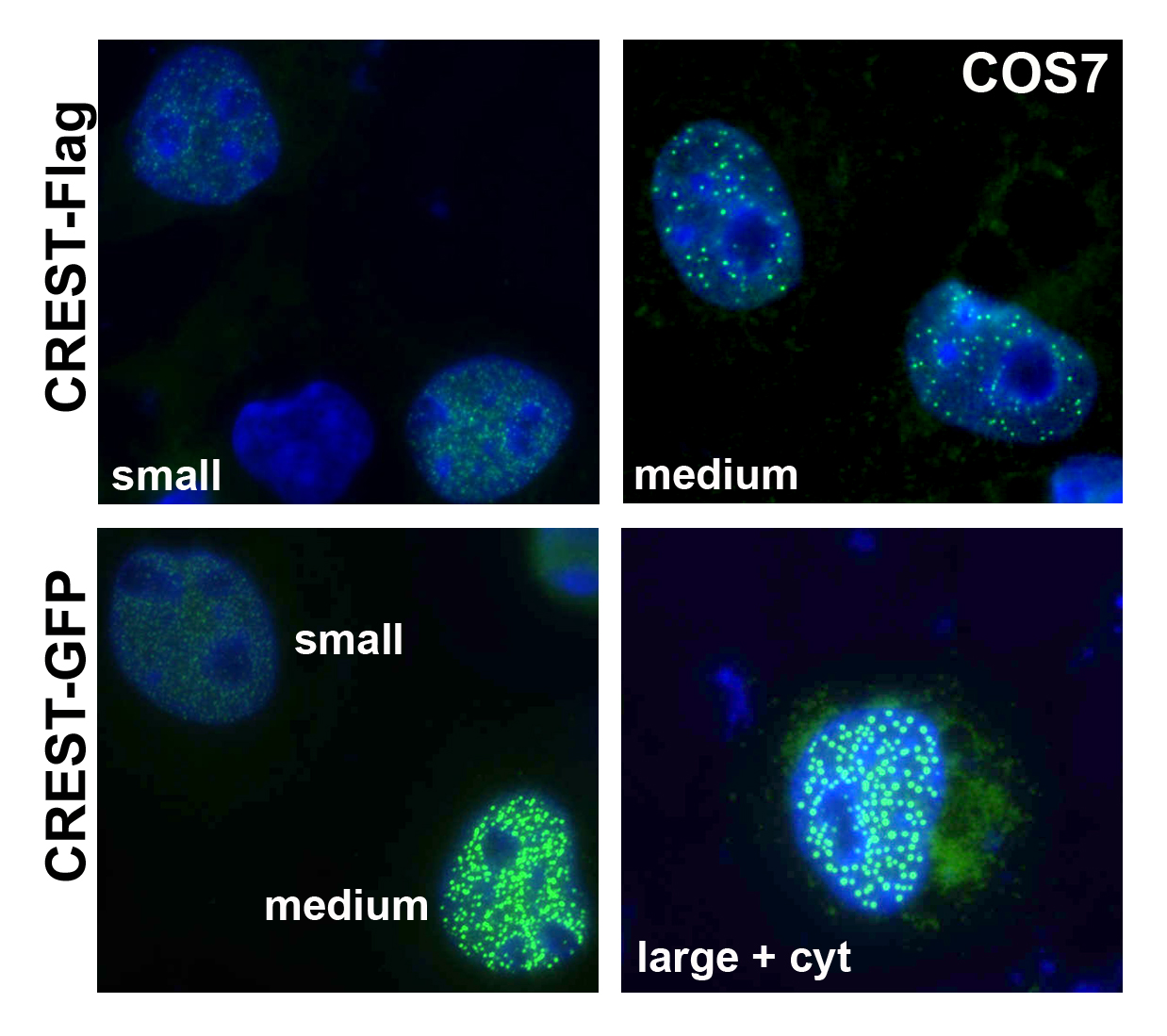

Supplement: Additional file 1: Figure S1. — Overexpressed CREST aggregates in a dose-dependent manner in COS7 cells. Flag- or GFP-tagged CREST protein displays diffuse and fine-granular distribution in the nucleus of low-expressing COS7 cells and forms dot-like aggregates as it accumulates. In cells with high levels of the protein, large nuclear aggregates together with cytoplasmic accumulation/aggregation are observed. Cells were analysed 24 hours post-transfection. Scale bar, 10 μm. [file 13024_2015_14_MOESM1_ESM.jpeg]

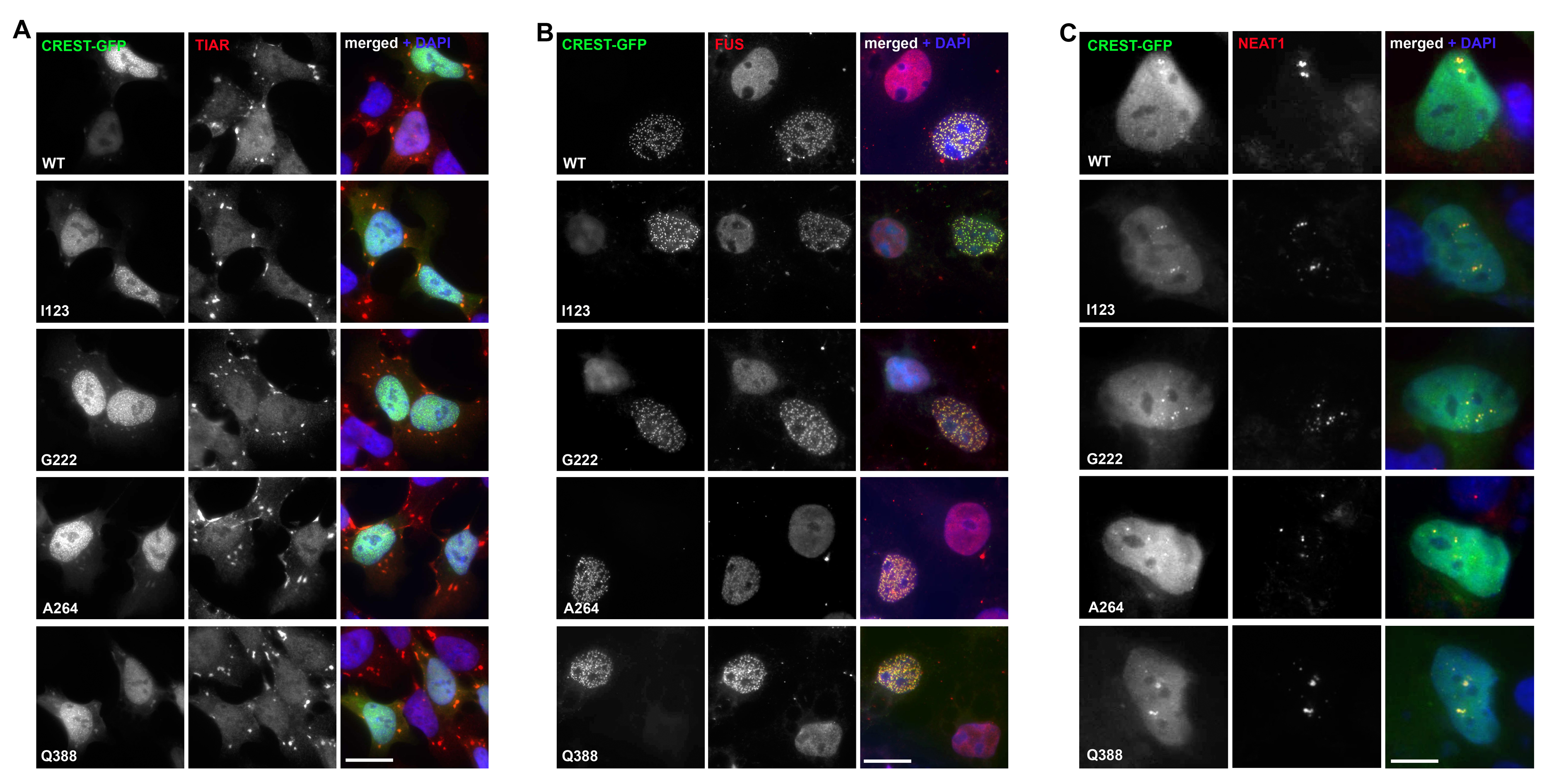

Supplement: Additional file 3: Figure S2. — ALS-linked CREST mutations do not alter the protein’s ability for stress granule or paraspeckle recruitment. (A) All CREST variants are recruited to sodium arsenite induced stress granules. (B) All CREST mutants sequester endogenous FUS protein into nuclear aggregates. (C) Mutations in CREST do not affect its enrichment in paraspeckles in low-expressing SH-SY5Y cells. Scale bars, A, B - 10 μm, C - 5 μm. [file 13024_2015_14_MOESM3_ESM.jpeg]

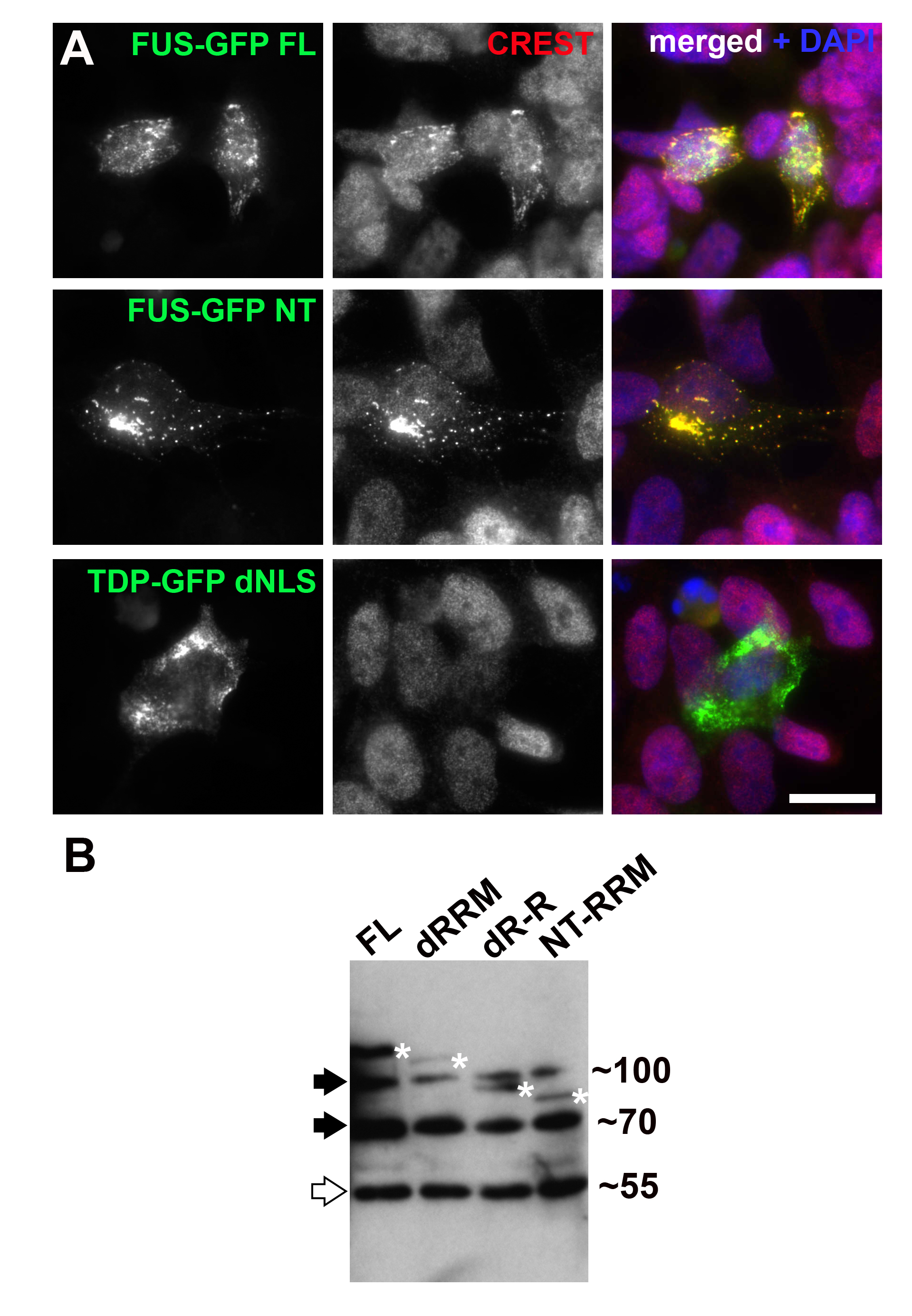

Supplement: Additional file 4: Figure S3. — Polyclonal anti-CREST antibody is cross-reactive to human FUS protein. (A) Aggregates formed by FUS-GFP R522G or FUS deletion mutant NT-RRM lacking C-terminal domains displayed high immunoreactivity with CREST antibody. Aggregates of GFP-tagged TDP-43 with deleted NLS were not recognised by this antibody ruling out possible cross-reactivity with GFP tag or non-specific recognition of aggregated protein species. (B) Full-length FUS and its deletion mutants are recognized by CREST antibody on Western blots. Specific bands corresponding to GFP-tagged FUS and its deletion mutants dRRM (lacking RRM), dR-R (lacking RRM and RGG3) and NT-RRM (lacking entire C-terminus, amino acids 360-526) are indicated with asterisks. Open arrow points to endogenous CREST protein and black arrows point to non-specific bands. Scale bar, 10 μm. [file 13024_2015_14_MOESM4_ESM.jpeg]

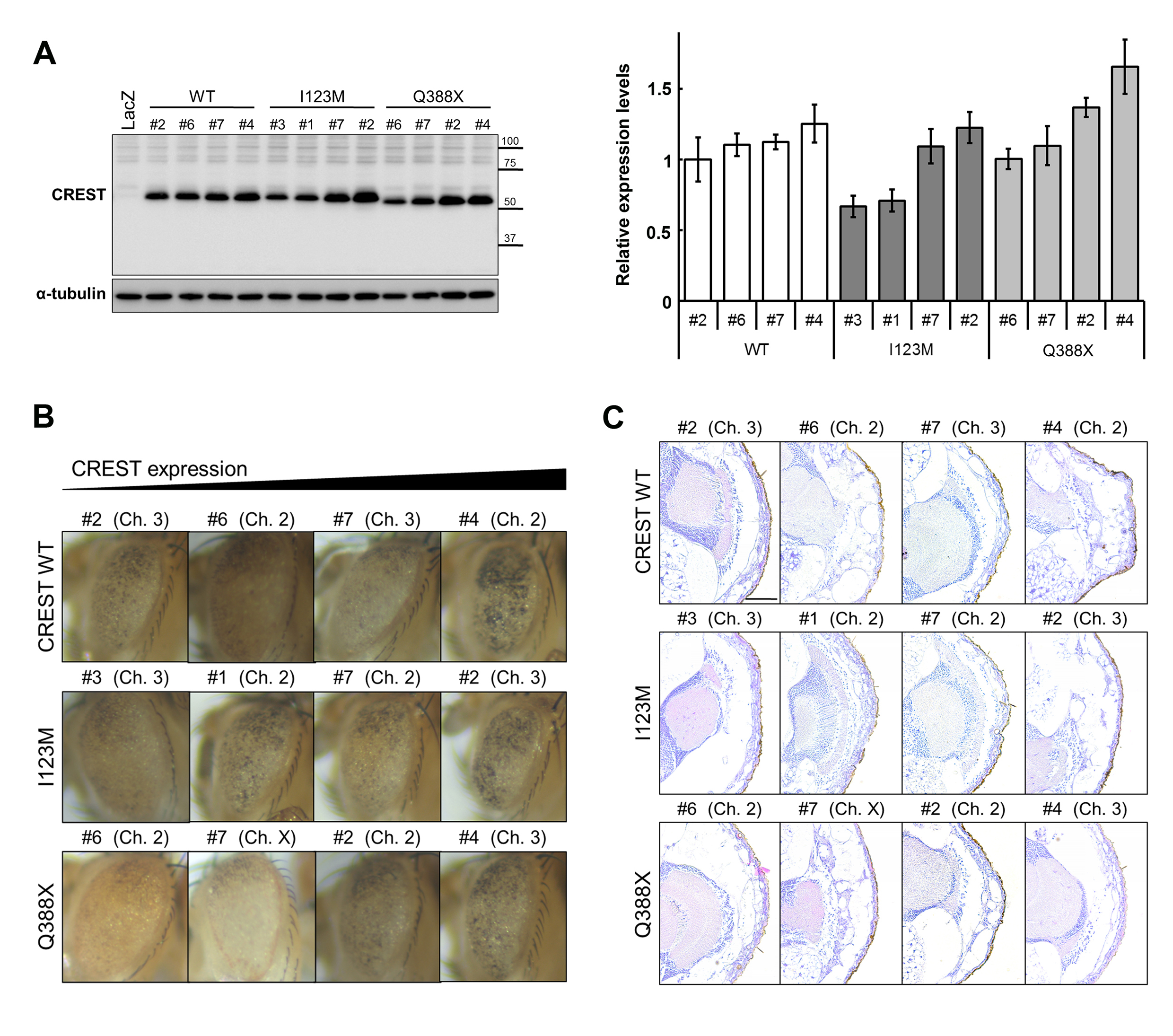

Supplement: Additional file 5: Figure S4. — CREST overexpression in Drosophila melanogaster retinal neurons results in retinal degeneration. (A) Transgenic fly lines expressing normal human CREST or its mutants I123M and Q388stop in retinal photoreceptor neurons with different levels of protein expression were generated. Protein levels were measured in the heads of transgenic flies by Western blotting using anti-CREST antibody and band intensities were quantified (bar chart shows mean ± s.d., CREST WT line #2 = 1.0). (B,C) Expression of wild-type or mutant CREST leads to severe retinal degeneration as evidenced by eye depigmentation (B) and its abnormal histology (C, H&E staining). The chromosome with transgene insertion (Ch) for each line is indicated. [file 13024_2015_14_MOESM5_ESM.jpeg]
